# Chicago College of Dental Surgery

**Published:** 1886-04

**Authors:** T. W. Brophy

**Affiliations:** Secretary.


### CHICAGO COLLEGE OF DENTAL SURGERY.

The fourth annual commencement exercises of the Chicago College of Dental Surgery were held at the First

Methodist Church, Chicago, on Wednesday afternoon, March 31st, 1886.

The class valedictory was delivered by Robert E. Moon, D. D. S., and the address to the graduates by W. L. Copeland, M. D., C. M., M. R. C. S., professor of Anatomy.

The number of matriculates for the session was eighty-one, an increase of thirty-one over the previous course.

The degree of D.D.S. was conferred on the following graduates by Dr. James A. Swasey, President of the Board of Directors.

| | |
|---|---|
| Harry Fenn Carson, - - | Illinois |
| Emory Melvil Cheadle, M. D., - | Oregon |
| Louis Clusmann, - - - | Illinois |
| Joseph Grant Emery, - - - | " |
| Gilbert Walter Eutsminger, - - | " |
| Frank Eshbaugh, - - - - | " |
| Ernst August Huxmann, - - - | " |
| Henry Frederick Marcoux, - - | " |
| Joseph Perry Mertes, - - | Wisconsin |
| Theodore Felix Molt, - - - | Illinois |
| Robert Ellsworth Moon, - - | Indiana |
| Otto Henry Staehle, - - - | Illinois |
| James Stewart, - - - - | " |
| Thomas Benton Wheeler, - - | " |
| Ellsworth Otis Whipple, - - | New York |
| Alfred Rogers Wilcox, - - | Illinois |

T. W. BROPHY, *Secretary.*

